# Evaluating knowledge and attitudes scales for the care of older adults among nursing students in Ghana

**DOI:** 10.1186/s12912-023-01195-y

**Published:** 2023-02-21

**Authors:** Diana Abudu-Birresborn, Sarah Brennenstuhl, Martine Puts, Lynn McCleary, Vida Yakong, Charlene H. Chu, Lisa Cranley

**Affiliations:** 1 Lawrence S. Bloomberg Faculty of Nursing, University of Toronto, 155 College Street, Suite 130, Toronto, ON M5T 1P8 Canada; 2 Department of Nursing, Brock University, 1812 Sir Isaac Brock Way, St. Catharine’s, ON L2S 3A1 Canada; 3 School of Nursing and Midwifery, University for Development Studies, Box 1350, Tamale, Ghana

**Keywords:** Knowledge, Attitudes, Older adults, Nursing students, Instrument evaluation, Ghana, Africa

## Abstract

**Background:**

Understanding nursing students’ knowledge about and attitudes toward older adults’ using context-specific survey instruments can help to identify and design effective learning and teaching materials to improve the care for persons 60 years and above. However, there are no validated instruments to examine nursing students’ knowledge and attitudes toward the care for older adults in the African context. The study aimed to evaluate the items on the Knowledge about Older Patients Quiz and Kogan's Attitudes towards Old People Scale suitable for the African context.

**Methods:**

A cross-sectional study was conducted using second-and third-year nursing students from two public Nursing Training Institutions in Ghana. Using Sahin’s rule of sample size estimate of at least 150 participants for unidimensional dichotomous scales, 170 nursing students were recruited to participate after an information session in their classrooms. Data were collected from December 2019—March 2020 using the Knowledge about Older Patients Quiz and Kogan’s Attitudes Towards Old People Scale. Item response theory was employed to evaluate the Knowledge about Older Patients Quiz difficulty level and discrimination indices. Corrected item-to-total correlation analysis was conducted for Kogan's Attitudes towards Old People Scale. The internal consistency for both scales was examined.

**Results:**

Of the 170 participants, 169 returned completed surveys. The mean age of participants was 21 years (SD = 3.7), and (54%) were female. Of the 30-items of the Knowledge about Older Patients Quiz, seven items were very difficult for most students to choose the correct response, and one was easy, as most of the students chose the correct response. Although 22 items demonstrated appropriate difficulty level, discrimination indices were used to select the final 15- items that discriminated moderately between upper and lower 25% performing students. The Kuder-Richardson-20 reliability was. 0.30, which was low. Considering Kogan's Attitudes towards Old People scale, 10-items were removed following negative and low corrected item-to-total correlation and a high Alpha coefficient if items were deleted. The final 22-items had a Cronbach alpha coefficient of 0.65, which was moderately satisfactory.

**Conclusion:**

Evaluation of the scales demonstrated essential content validity and moderate internal consistency for the context of our study. Further research should focus on ongoing context-specific refinement of the survey instruments to measure nursing students’ knowledge about and attitudes toward caring for older adults in the African context.

**Supplementary Information:**

The online version contains supplementary material available at 10.1186/s12912-023-01195-y.

## Background

The population of persons 60 years and older is growing in the African region, like other regions [1]. It is projected that between 2020 and 2050, the number of older adults 60 + years is expected to triple from 74.4 million to 235.1 million with about two-third of African countries being home for about one million person's 60 + years [2, 3]. In Ghana, the 2021 population and housing census report indicate that there are almost two million person's (1,991, 736) 60 + years representing 6.5% of the total population [4]. The increasing proportion of older adults will likely correspond to increased health and nursing care utilization. Although there are no existing data to quantify the rate and health care utilization trends by older adults in Ghana, previous scholars have identified several factors associated with health care utilization by older adults [5]. These factors include increasing age, availability of health insurance and access to healthcare, and multiple complex chronic conditions such as cardiovascular diseases, stroke, diabetes, hypertension, and cognitive impairment, [5–7]. Some of these factors, such as multiple complex chronic conditions, result in older adults presenting with multiple geriatric syndromes, including delirium, incontinence, frailty and falls [8]. These complex chronic conditions, coupled with complications, require nurses with sufficient gerontological nursing knowledge to provide adequate and quality nursing care for older adult patients [9].

Evidence suggests that nursing students show little interest in working with older adults for reasons such as heavy workloads and insufficient knowledge in gerontology [10]. Nursing students’ ability to demonstrate their understanding of gerontological nursing content through their responses to knowledge items or tests can provide insight into their competencies in caring for older adults [11]. It is necessary to examine nursing students’ knowledge and attitudes towards older adults to identify opportunities and gaps to facilitate nursing students’ preparation in the care of older adults in Africa. However, no validated instruments exist to examine nursing students’ knowledge and attitudes towards older adults in the African context.

There is no specialized gerontological nursing care for older adult patients in Ghana, due to the lack of post-basic specialty programs in gerontology [12]. In addition, there are no public long-term care facilities or nursing homes for older adults. Older adults are cared for by generalist nurses in emergency departments and medical-surgical wards along with the general population. It is imperative to enhance nursing students’ theoretical knowledge and skills to ensure quality care for older adults. Nursing students with high theoretical knowledge and positive attitudes, with or without the preference for working with older adults are likely to demonstrate adequate competencies in providing care for older adult patients [10, 13]. Various instruments have been developed and validated for assessing nursing students’ preparation, such as knowledge and attitudes scales.

The most widely used survey instruments are the Facts on Ageing Quiz (FAQ) and Kogan’s Attitudes towards Old People (KAOP) scale [14, 15]. The Knowledge about Older Patients Quiz (KOP-Q) [16] was recently developed and validated to measure knowledge in acute care settings in the Netherlands. Until now, the KOP-Q has been used to measure nurses’ knowledge about older patients in general hospitals in the Netherlands and USA [9]. To our knowledge, the KOP-Q has not been used among nursing students in Ghana. Another commonly used is Kogan’s Attitudes towards Old People (KAOP) scale [14] which has been widely used with a reported Cronbach alpha of 0.70 among nursing students in Asia, [17, 18], middle East [19, 20] and a correlation coefficient of 0.71 in Africa [21]. However, Faronbi, Adebowale [21], who used it in Nigeria, used only nine items of the scale. The authors did not provide a rationale for using the nine items. A probable assumption could be that the nine items were context-specific and contributed strongly to the overall coefficient of their survey instrument.

In the current study, the KOP-Q and KAOP were adapted for use with Ghanaian nursing students. They did not require translation since English is the official language of instruction. However, questions about how suitable these survey instruments are to the African context remain. This paper aimed to evaluate the suitability of the KOP-Q and the KAOP scale for the African context focusing on item difficulty and discrimination for the KOP-Q and corrected item-to-total correlation for the KAOP scale. This study is part of a larger mixed-method research project that examined the learning experiences of nursing students to care for older adults in Ghana [22].

## Methods

### Study design and setting

A cross-sectional survey was conducted using a convenience sample [23] of nursing students. Convenient sampling was appropriate because of the feasibility of our study. First, nursing students were accessible as they had completed their clinical rotation and were back in school [24]. Second, we had limited time for recruitment as this study was part of a Ph.D. dissertation. Using convenient sampling can be helpful in our exploratory study to obtain a foundational range of attitudes and gaps in knowledge that can further be explored rigorously [25]. Although we used convenient sampling, nursing students were selected based on the northern-southern geographical categorization of Ghana to provide a representative sample. Nursing students were selected from two Public Nursing and Midwifery Training Colleges attached to Teaching hospitals from the northern and southern sectors of Ghana. The northern sector is primarily Muslim-dominated and share ethnic and cultural traditions which are slightly different from the ethnic and cultural traditions of the Christian-dominated southern sectors of Ghana. Second- and third-year nursing students were invited to participate in the study because they had more clinical experience than first-year students, which was relevant for answering the questionnaires.

### Sample

The sample size was calculated based on our main study [22]. We employed *N* = 50 + 8 k, where *N* = sample size, and *k* = the number of predictors and covariates [26]. The predictors and covariates included knowledge, attitudes, age, gender, years of school, region, religion and living with/lived with an older adult. Thus, *N* = 50 + 8(8) = 114 nursing students. However, previous researchers [27, 28] reported a 70% response rate among nursing students. With an estimated 70% response rate, the sample size was 114/70 × 100 = 163. Due to uncertainties such as incomplete printing, and misplaced survey documents by students, and to reduce the impact of possible dropouts, the sample size was rounded to 170 nursing students.

According to Sahin’s rule of sample size estimates for unidimensional dichotomous scales, at least 150 participants are adequate to determine item parameters accurately [29]. Therefore, the 170 nursing students in our original sample estimate were adequate and appropriate for analysis in the current study.

### Procedure

Data were collected using survey questionnaires between December 2019—March 2020. An information session by the principal investigator (DAB) was conducted in the second-and third-year classrooms. An envelope that contained the questionnaires, information sheet and consent form, invitation to participate in the second phase of our study and a gift card equivalent to CAD $5 (GH Cedis 15) as a token of appreciation was given to participants who volunteered. The questionnaire had four sections. Section A focused on the sociodemographic data; Section B included the KOP-Q, Section C included the KOAP scale, and Section D was the Self-efficacy to Care for Older Adults (GES-COA) scale. Sections A, B and C were the focus of this paper. The survey instruments were pilot tested to determine nursing students understanding of the items and estimate how long it took to complete the questionnaires. The results of the pilot testing were not included in the final analysis. The estimated time for completing sections A, B and C of the survey was 25 min. The survey was provided on paper, and participants were allowed to submit the completed survey within one week. The questionnaires were administered in English. Completed questionnaires were either dropped in a sealed box that was placed at the reception desk at the school or handed to the principal investigator in a sealed envelope onsite.

### Measures

The Knowledge about Older Patients Quiz (KOP-Q) [9, 16] is a unidimensional scale that consists of 30 true or false items to measure nursing students’ knowledge in the care of older adults. The KOP-Q examines nurses’/nursing students’ knowledge of the normal ageing process, depression and delirium in older adults, geriatric syndromes, nutrition and fluid imbalance, polypharmacy, referrals, and family caregivers’ support. A correct response scored + 1, and an incorrect response scored zero. The scores are summed for a total score. Face validity of 0.91 and construct validity of 0.70 have been reported [16, 30] in the Netherlands and USA. As a validated tool and upon review by experts of gerontological nursing education and survey methodology from our research team, the face and construct validity scores were deemed adequate. One of the experts and the first author are Ghanaians, who evaluated the items for contextual appropriateness. The items had appropriate language and substantive content levels to measure knowledge about the care for older adults in our study context.

The Kogan’s Attitude Towards Old People Scale (KAOP) [14] consists of 32- item statements. The items of the KAOP scale focused on issues such as the housing arrangements of older adults, the environment and personal appearance, interpersonal relationships with other age groups and dependence of older adults. Item responses are rated on a 5-point Likert scale, ranging from 1 strongly disagree to 5 strongly agree. Half of the statements are negative and the other half are positive regarding attitudes toward older individuals [31]. Negative statements were reverse coded so that there is one total positive score ranging between 32–160. A neutral response score of 3 was used to determine the maximum neutral response of 96 as the cut-off point. Higher scores above 96 indicated positive attitudes, and scores lower than 96 indicated negative attitudes. In addition to the two scales, participants’ socio-demographic data were collected to describe the sample. These included age, gender, years of school (second /third year), living with/having lived with an older adult, religion, and region (southern /northern sector).

## Statistical analysis

All analyses were conducted using SPSS (IBM version 26).

### Analysis of KOP-Q

Item difficulty and discrimination indices were calculated as follows. First, the participant knowledge scores were summed, and the total knowledge scores were calculated. Next, data were categorized in ranks from the lowest to the highest scores. Participants were categorized into quartiles; the lowest 25%, the middle 50% and the highest 25%. The lowest 25% was calculated using the total number of correct answers in the quartile and divided by the number of participants in that quarter (42 participants). The highest 25% was calculated using the total number of correct answers in the highest 25% divided by the number of participants in the quarter (42 participants). Participants within the lowest and highest, 25% were used for the discrimination indices [32, 33].

Item difficulty is the percentage of participants who answered an item correctly. Item difficulty was calculated using the formula:

$$\mathrm{ID}= \frac{\mathrm{number\,of\,students\,who\,answered\,an\,item\,correctly}}{\mathrm{ total\,number\,of\,participants }}\mathrm{x }100$$

The score ranges between 0–100% [34]. The lower the difficulty level, the harder the items, suggesting fewer students chose the correct response. The higher the difficulty level, the easier the items, indicating a majority of the students chose the correct response [32, 33].

Optimal item difficulty levels are somewhat higher than halfway between guessing and students actually knowing the correct response to an item [32]. For dichotomous (true/false) response choice questions, optimal difficulty ranges between 30–85%. Items less than 30% are considered too difficult, and more than 85% are too easy and recommended to be removed or revised [35].

However, for the selection and examination of final items for the psychometric property analysis of the scale, discrimination indices (DI) were considered [36]. The discrimination index (DI) was calculated using the formula: [37]

$$\text{DI}\, = \text{Proportion}\;\text{of}\;\text{participants}\;\text{with}\;\text{highest}\;25\%\;\text{score} - \;\text{proportion}\;\text{of}\;\text{participants}\;\text{with}\;\text{the}\;\text{lowest}\;25\%\;\text{score}$$

Various factors, such as the number of items, unidimensionality of the scale and item difficulty, impact the DI threshold of items [37]. A discrimination index threshold of ≥ 0.2 is recommended to determine items to retain or remove [37, 38]. Using our content judgement, some items with a DI of 0.19 were approximating 0.2 and included for analysis [37]. The DI calculations were repeated after items were removed one after the other which enhanced the DI for the items retained. The Kuder Richardson (KR20) reliability for the retained items was calculated. KR20 is the dichotomous equivalent of the Cronbach alpha coefficient and is appropriate for use with survey instruments that measure dichotomous items such as true/false or yes/no responses [39]. A KR20 of 0.70 and above is considered acceptable [40].

### Analysis of the KAOP scale

To evaluate and improve the internal consistency of KAOP, we calculated the corrected item-to-total correlations and Cronbach alpha if an item was deleted. Corrected item-to-total indicates how much each item correlates with the overall scale [41]. Negative and very low corrected item-to-total correlation suggest that items do not correlate adequately with the overall scale and should be removed [42]. Negative and very low corrected-item-to-total correlations were deleted one after the other; the deletions increased the overall Cronbach alpha of the scale [41, 43].

The extent and pattern of missing scale item values were examined using descriptive statistics; 95.8% of the sample provided complete data at the item level. With such a small proportion of missing data, complete case analysis was employed. The complete case analysis based on the amount of missing data did not impact the statistical efficiency due to the minimum required sample estimates [29].

## Results

### Characteristics of participants

Of the 170 questionnaires distributed, 169 were returned (99%). Participants had a mean age of 21 years (SD = 3.7) years. Of the 169 participants, 54% were female, and more than half (64%) of the participants were Christians. The majority (83%) of participants lived with or were currently living with an older adult. About half (49.1%) of the participants were in the second year of their nursing program, and the other half were in their third year.

### KOP- Q

Overall, 22 of the 30 original items had a difficulty level between 30–85% indicating optimum level (Supplementary Table 1). Seven items had item difficulty levels between 7.10% -28.40%, indicating very difficult items. Item SB02 *(For older people, bed rest is important to enhance recovery)* had a difficulty level of 92.6%, indicating that it was an easy item (Supplementary Table 1). Although eight items demonstrated very difficult or easy levels, four of the items; SB09, SB28, SB19 and SB21, were retained based on their discrimination index levels [38] (Supplementary Table 2).

For the discrimination indices of the original 30-items, 15 items discriminated moderately (0.24–0.50) between the upper 25% performing students and the lower 25% performing students and were retained. The total knowledge scores for the 15-items ranged between 0–15. The KR20 reliability was 0.30, which is considered low [44]. The final 15 items are presented in Supplementary Table 3.

### KAOP Scale

Of the 32-items, 10- items were removed due to low or negative corrected item-to-total correlations. The items did not contribute satisfactorily to the internal consistency of the scale based on the Cronbach alpha if an item is deleted (Supplementary Table 4). Further removal of items did not increase the Cronbach alpha coefficient. The final 22-items had a total score range between 22–110, with a Cronbach alpha coefficient of 0.65 and were considered moderately satisfactory [44] (Supplementary Table 5).

## Discussion

This paper aimed to evaluate the suitability of the KOP-Q and the KAOP scale for the African context focusing on item difficulty and discrimination for the KOP-Q and corrected item-to-total correlation for the KAOP scale. This study showed that 15-items of the original 30-items of the KOP-Q were suitable for the Ghanaian context, of which four items demonstrated low difficulty levels.

The four items examined topics such as distinguishing signs of depression from normal ageing processes, fluid and nutritional imbalance in older adults, effects of medication on geriatric syndromes and family caregiving support services. These topics are either not in the current gerontological nursing curriculum and are not taught or are inadequately taught which may have accounted for the low difficulty levels of the items among Ghanaian nursing students [45]. The outcome was not surprising for the item on family caregivers’ support services. In Ghana, family caregiving is considered the traditional responsibility of adult children, and other family members which has little to no support services from the government and other agencies [46]. As a result, the topic of family caregiving support services may be missing from and not taught in the gerontological nursing curriculum in Ghana. The low difficulty levels of the items highlight a broader gap in gerontological nursing education in the Ghanaian context and can place older adults at a greater risk for poorer outcomes, including longer lengths of hospital stay, increased readmission rates, increased morbidity, and mortality [47, 48]. Thus, there is a need to reinforce nursing students’ gerontological nursing education to enhance their readiness to meet the complex and demanding needs of older adults [10, 49].

A high percentage of nursing students correctly responded to an item that examined bedrest and recovery in older adults demonstrating the item was a very easy item. It is generally known that bedrest enhances recovery in older adults and is likely the reason for the high percentage score of the item. Very high or low difficulty items make it challenging for assessors to determine which students know the content and those who do not and resort to guessing [50]. The final items that met both difficulty and discriminatory qualities and measure substantive gerontological nursing topics/areas were retained using discrimination indices.

Our study also showed that 22 -items of the original 32 items of the KAOP scale would be applicable to our geographical context. Ten items showed redundancy and did not contribute significantly to the internal consistency of the scale. The remaining 22-items demonstrated moderate internal consistency, which is acceptable for analysis. The moderate internal consistency is similar to the findings of a study conducted to assess the validity of the KAOP scale in Thailand, Myanmar, and Indonesia, where moderate internal consistency of the scale was reported [18]. Researchers have maintained that the number of items of a scale contributes to the internal consistency [51], which may have accounted for the internal consistency of the KAOP scale in this study. In addition, sociocultural variations in the care for older adults may have contributed to the moderate internal consistency. The original scale was developed in the United States, where the perceived individualist culture influences the values of caring for older adults like other western cultures compared to the collective culture of caring for older adults in Ghana [52, 53].

The low internal consistency of the KOP-Q was not surprising because knowledge tests such as this are often very broad in content and measure multiple theoretical, conceptual, and clinical aspects of a topic. This can result in heterogenous and unrelated items and subsequently a low internal consistency [44, 54, 55]. The KR20 reliability is influenced by the difficulty levels, the number of items and the variance of the overall scores [51, 56]. Increasing the number of items and students’ content knowledge about the difficult topics through effective teaching and learning designs can refine the KOP-Q for the African context [14, 51, 56].

### Strengths and limitations

This study was an exploratory study due to the lack of prior research. Hence, we adapted and evaluated existing validated survey instruments. The adaptation resulted in the reduction of items due to a high correlation with other items on the scales. The number of items, in addition to the sample size estimate for analysis, may have influenced the findings of the scales, especially the KOP-Q [14]. Although the sample size estimate meets the minimum requirement to accurately determine item parameters for unidimensional dichotomous scales, a larger sample size estimate may improve the scales psychometric properties. Nonetheless, there was a high response rate despite the low KR20 reliability. Using the KOP-Q scale may allow educators and researchers to identify the different levels of performance on specific topics or areas and provide an overview of issues that may need to be reinforced or introduced into gerontological nursing content. Decisions about the items were made based on the substantive content concepts in the care of older adults in acute care settings, which discriminated well among students with high and low knowledge about care of older adults.

Another limitation to note was that the original scale was developed and tested among registered nurses; however, the scale was used among nursing students in this study who may not have had similar education and experience levels as the nurses in the original study. This may have accounted for the low difficulty levels and discrimination indices of the items. Furthermore, we could not examine other psychometric properties, such as test–retest reliability. The dichotomous items of the KOP-Q scale likely increased guessing among students, which may have impacted the outcome of the findings. Therefore, future researchers could consider revising the scoring from dichotomous to Likert scale to reduce the likelihood of guessing. In addition, the self-reported scales may induce social desirability bias due to the sociocultural expectations of caring for older adults which may have contributed to the findings of the KAOP. Thus, the results should be interpreted with caution.

### Implications for nursing education, practice, and research

The present study has the potential to inform nursing education by highlighting nursing students’ difficulties and gaps in fundamental topics in gerontological nursing education. The findings may help to recognize pitfalls between what is taught in class and the clinical setting and help nurse educators make effective decisions to bridge the theory- practice gap to improve the quality of care for older adults. In addition, identifying knowledge deficits can facilitate the development of innovative and effective teaching strategies to promote and enhance positive gerontological learning experiences. The study highlights the potential to improve practice through continuing career development programs to bridge the fundamental knowledge gaps in gerontological nursing care. Conducting a needs assessment using the revised scales can help to identify practicing nurses’ knowledge deficits about specific topics in the care of older adults that may require training to improve positive outcomes. The present study also underscores the importance of using substantive content and rigorous methods in research to ensure survey instruments are valid, appropriate, and context-specific to test students’ knowledge. Further testing and adaptation of the KOP-Q should be conducted in LMICs to ensure the psychometric properties are consistent using large samples, especially for the African region.

## Conclusion

Preparing nursing students to face the changing demands of care due to the increasing older adult patient population in acute care hospitals is crucial in lower- and middle-income countries (LMICs), including Ghana. Effective evaluation of knowledge acquired in the care of older adults with validated survey instruments that are context-specific and recognizing the sociocultural values of caring for older adults is essential. This can facilitate the evaluation, revision and enhance gerontological nursing content to improve nursing students’ readiness to care for older adults. The revised KOP-Q and the KAOP can provide foundational estimates that can be potentially useful for examining and identifying deficits in knowledge and attitudes toward the care of older adults. The scales demonstrated substantive content and acceptable internal consistency. Future research should consider the development and psychometric testing of instruments in the care of older adults specific to the African context.

## Supplementary Information

**Additional file 1:** **Table 1.** Item Discrimination analysis. **Table 2.** Item with extreme easy and difficulty levels. **Table 3. **Final items retained for KOP-Q. **Table 4.** Items removed for KAOP.** Table 5.** Final items of KAOP.

## Data Availability

The datasets used and/or analyzed during the current study are not publicly available because the datasets are part of a larger study which contains confidential material but are available from the corresponding author on reasonable request.
