# Supplementary material for: Evaluating knowledge and attitudes scales for the care of older adults among nursing students in Ghana
Source: BMC Nurs. 2023 Feb 21;22:47. doi: 10.1186/s12912-023-01195-y (PMC9942360; doi:10.1186/s12912-023-01195-y)
Supplement: Supplementary file 1 — Additional file 1: Table 1. Item Discrimination analysis. Table 2. Item with extreme easy and difficulty levels. Table 3. Final items retained for KOP-Q. Table 4. Items removed for KAOP. Table 5. Final items of KAOP. [file 12912_2023_1195_MOESM1_ESM.docx]

**Supplementary Material**

**Table 1: Item Discrimination analysis**

| **No.** | **Item** | **Correct total** | **Item difficulty** | **P** | **Q** | **PQ** | **Lower group** | **Upper group** | **Discrim. Index** | **Remove/**  **Retain** |
| --- | --- | --- | --- | --- | --- | --- | --- | --- | --- | --- |
| 1 | Forgetfulness, concentration problems, and indecisiveness are parts of aging rather than indicators of depression | 17 | 10.06 | 0.10 | 0.90 | 0.09 | 0.12 | 0.14 | 0.02 | Remove |
| 2 | For older people, bed rest is important to enhance recovery | 157 | 92.90 | 0.93 | 0.07 | 0.07 | 0.93 | 0.95 | 0.02 | Remove |
| 3 | Individuals with a cognitive disorder, such as dementia, are at greater risk for delirium | 125 | 73.96 | 0.74 | 0.26 | 0.19 | 0.60 | 0.81 | 0.21 | Retain |
| 4 | In general, older people are more sensitive to medication because their kidney and liver functions are declining. | 140 | 82.84 | 0.83 | 0.17 | 0.14 | 0.71 | 0.93 | 0.21 | Retain |
| 5 | People rarely remember that they were anxious or restless during delirium | 128 | 75.74 | 0.76 | 0.24 | 0.18 | 0.64 | 0.81 | 0.17 | Remove |
| 6 | In the case of delirium, bright lighting should always be used to illuminate all of the corners of the room | 54 | 31.95 | 0.32 | 0.68 | 0.22 | 0.19 | 0.50 | 0.31 | Retain |
| 7 | In the case of delirium, activities should be spread out evenly over the day | 53 | 31.36 | 0.31 | 0.69 | 0.22 | 0.24 | 0.43 | 0.19 | Retain |
| 8 | Depression is recognized in older people less frequently than it is in younger people | 73 | 43.20 | 0.43 | 0.57 | 0.25 | 0.19 | 0.67 | 0.48 | Retain |
| 9 | In the case of depression, memory problems may occur | 31 | 18.34 | 0.18 | 0.82 | 0.15 | 0.05 | 0.31 | 0.26 | Retain |
| 10 | It is good to provide extensive instruction about how to complete tasks to individuals with apraxia | 143 | 84.62 | 0.85 | 0.15 | 0.13 | 0.76 | 0.88 | 0.12 | Remove |
| 11 | Pressure that cuts off the blood supply to tissue for two hours may result in pressure ulcers | 119 | 70.41 | 0.70 | 0.30 | 0.21 | 0.71 | 0.86 | 0.14 | Remove |
| 12 | Identify pressure ulcers only if blister formation or abrasions have occurred. | 71 | 42.01 | 0.42 | 0.58 | 0.24 | 0.29 | 0.43 | 0.14 | Remove |
| 13 | Stress incontinence may occur in people who are not capable of unzipping/ unbuttoning their trousers or skirts | 71 | 42.01 | 0.42 | 0.58 | 0.24 | 0.26 | 0.60 | 0.33 | Retain |
| 14 | Unexpected urinary incontinence in an older person may indicate that the person has a urinary tract infection | 64 | 37.87 | 0.38 | 0.62 | 0.24 | 0.26 | 0.69 | 0.43 | Retain |
| 15 | Incontinent individuals must have their soiled clothing changed but do not need to be placed on the toilet afterward. | 124 | 73.37 | 0.73 | 0.27 | 0.20 | 0.69 | 0.83 | 0.14 | Remove |
| 16 | Malnutrition can have negative effects thinking and memory | 12 | 7.10 | 0.07 | 0.93 | 0.07 | 0.07 | 0.14 | 0.07 | Remove |
| 17 | An older person with a body mass index greater than 25 kg/m2 cannot be undernourished | 81 | 47.93 | 0.48 | 0.52 | 0.25 | 0.38 | 0.64 | 0.26 | Retain |
| 18 | Older people need less fluid because they exercise less. | 25 | 14.79 | 0.15 | 0.85 | 0.13 | 0.12 | 0.26 | 0.14 | Remove |
| 19 | It is good to have older people drink more often because they have a reduced thirst sensation | 28 | 16.57 | 0.17 | 0.83 | 0.14 | 0.10 | 0.31 | 0.21 | Retain |
| 20 | Lowering the frequency of a medication is an effective intervention to achieve (medication) adherence by patients | 100 | 59.17 | 0.59 | 0.41 | 0.24 | 0.40 | 0.60 | 0.19 | Retain |
| 21 | Medication may cause geriatric problems such as memory deficits, incontinence, falling, and depression | 48 | 28.40 | 0.28 | 0.72 | 0.20 | 0.17 | 0.43 | 0.26 | Retain |
| 22 | In the case of difficulty swallowing, all medicines must be ground to ensure that patients ingest them. | 61 | 36.09 | 0.36 | 0.64 | 0.23 | 0.31 | 0.43 | 0.12 | Remove |
| 23 | Pain medication should be administered to older people as little as possible because of the possibility of addiction. | 125 | 73.96 | 0.74 | 0.26 | 0.19 | 0.74 | 0.83 | 0.10 | Remove |
| 24 | Risk of falling is higher for people in the hospital setting than those living at home. | 130 | 76.92 | 0.77 | 0.23 | 0.18 | 0.74 | 0.83 | 0.10 | Remove |
| 25 | Asking an individual whether he or she has fallen in the past 6 months is a good way of assessing for risk of falling. | 114 | 67.46 | 0.67 | 0.33 | 0.22 | 0.52 | 0.83 | 0.31 | Retain |
| 26 | Meeting with families during patient assessment is required only for persons with dementia | 119 | 70.41 | 0.70 | 0.30 | 0.21 | 0.71 | 0.81 | 0.10 | Remove |
| 27 | Overburdening of family caregivers may lead to abuse of the person for whom they are providing care. | 113 | 66.86 | 0.67 | 0.33 | 0.22 | 0.52 | 0.81 | 0.29 | Retain |
| 28 | Most family caregivers do not need additional support from community health nurses | 35 | 20.71 | 0.21 | 0.79 | 0.16 | 0.10 | 0.29 | 0.19 | Retain |
| 29 | As a nurse, you have to speak clearly into the ear of a hearing-impaired older adult | 135 | 79.88 | 0.80 | 0.20 | 0.16 | 0.67 | 0.93 | 0.26 | Retain |
| 30 | When speaking to hearing-impaired older adults, it is best to speak at normal volume | 99 | 58.58 | 0.59 | 0.41 | 0.24 | 0.60 | 0.64 | 0.05 | Remove |

*P= Proportion of students who had correct responses

*Q= Proportion of students who had incorrect responses

**Table 2. Item with extreme easy and difficulty levels**

| **No.** | **Item** | **Correct total** | **Item difficulty** | **Lower group** | **Upper group** | **Discrim.**  **Index** |
| --- | --- | --- | --- | --- | --- | --- |
| SB01 | Forgetfulness, concentration problems, and indecisiveness are parts of aging rather than indicators of depression | 17 | 10.06 | 0.12 | 0.14 | 0.02 |
| SB02 | For older people, bed rest is important to enhance recovery | 157* | 92.90 | 0.93 | 0.95 | 0.02 |
| SB16 | Malnutrition can have negative effects thinking and memory | 12 | 7.10 | 0.07 | 0.14 | 0.07 |
| SB18 | Older people need less fluid because they exercise less. | 25 | 14.79 | 0.12 | 0.26 | 0.14 |
| SB09 | In the case of depression, memory problems may occur | 31 | 18.34 | 0.05 | 0.31 | 0.26 |
| SB28 | Most family caregivers do not need additional support from community health nurses | 35 | 20.71 | 0.10 | 0.29 | 0.19 |
| SB19 | It is good to have older people drink more often because they have a reduced thirst sensation | 28 | 16.57 | 0.10 | 0.31 | 0.21 |
| SB21 | Medication may cause geriatric problems such as memory deficits, incontinence, falling, and depression | 48 | 28.40 | 0.17 | 0.43 | 0.26 |

SB= Section B of questionnaire

*= low difficulty level (Easy item)

**Table 3: Final items retained for KOP-Q**

| **No.** | **Item** | **Correct total** | **Item difficulty** | **P** | **Q** | **PQ** | **Lower group** | **Upper group** | **Discrim. Index** | **Retain/**  **Remove** |
| --- | --- | --- | --- | --- | --- | --- | --- | --- | --- | --- |
| 3 | Individuals with a cognitive disorder, such as dementia, are at greater risk for delirium | 125 | 73.96 | 0.74 | 0.26 | 0.19 | 0.48 | 0.93 | 0.45 | Retain |
| 6 | In the case of delirium, bright lighting should always be used to illuminate all of the corners of the room | 54 | 31.95 | 0.32 | 0.68 | 0.22 | 0.17 | 0.52 | 0.36 | Retain |
| 7 | In the case of delirium, activities should be spread out evenly over the day | 53 | 31.36 | 0.31 | 0.69 | 0.22 | 0.12 | 0.57 | 0.45 | Retain |
| 8 | Depression is recognized in older people less frequently than it is in younger people | 73 | 43.20 | 0.43 | 0.57 | 0.25 | 0.21 | 0.64 | 0.43 | Retain |
| 9 | In the case of depression, memory problems may occur | 31 | 18.34 | 0.18 | 0.82 | 0.15 | 0.12 | 0.38 | 0.26 | Retain |
| 13 | Stress incontinence may occur in people who are not capable of unzipping/ unbuttoning their trousers or skirts | 71 | 42.01 | 0.42 | 0.58 | 0.24 | 0.29 | 0.52 | 0.24 | Retain |
| 14 | Unexpected urinary incontinence in an older person may indicate that the person has a urinary tract infection | 64 | 37.87 | 0.38 | 0.62 | 0.24 | 0.24 | 0.69 | 0.45 | Retain |
| 17 | An older person with a body mass index greater than 25 kg/m2 cannot be undernourished | 81 | 47.93 | 0.48 | 0.52 | 0.25 | 0.29 | 0.64 | 0.36 | Retain |
| 19 | It is good to have older people drink more often because they have a reduced thirst sensation | 28 | 16.57 | 0.17 | 0.83 | 0.14 | 0.12 | 0.38 | 0.26 | Retain |
| 20 | Lowering the frequency of a medication is an effective intervention to achieve (medication) adherence by patients. | 100 | 59.17 | 0.59 | 0.41 | 0.24 | 0.43 | 0.74 | 0.31 | Retain |
| 21 | Medication may cause geriatric problems such as memory deficits, incontinence, falling, and depression | 48 | 28.40 | 0.28 | 0.72 | 0.20 | 0.17 | 0.52 | 0.36 | Retain |
| 25 | Asking an individual whether he or she has fallen in the past 6 months is a good way of assessing for risk of falling | 114 | 67.46 | 0.67 | 0.33 | 0.22 | 0.43 | 0.93 | 0.50 | Retain |
| 27 | Overburdening of family caregivers may lead to abuse of the person for whom they are providing care | 113 | 66.86 | 0.67 | 0.33 | 0.22 | 0.45 | 0.81 | 0.36 | Retain |
| 28 | Most family caregivers do not need additional support from community health nurses | 35 | 20.71 | 0.21 | 0.79 | 0.16 | 0.12 | 0.36 | 0.24 | Retain |
| 29 | As a nurse, you have to speak clearly into the ear of a hearing-impaired older adult | 135 | 79.88 | 0.80 | 0.20 | 0.16 | 0.67 | 0.98 | 0.31 | Retain |

*P= Proportion of students who had correct responses

*Q= Proportion of students who had incorrect responses

**Table 4. Items removed for KAOP**

| **No.** | **Items Removed** | **Corrected item-total**  **Correlations** | **Cronbach Alpha if item deleted** |
| --- | --- | --- | --- |
| SC29 | Most older adults are constantly complaining about the young generation’s behaviour | -0.181 | 0.581 |
| SC01 | It would be better if most older adults lived in housing with people of their age | -0.50 | 0.598 |
| SC19 | If older adults expect to be liked, they should eliminate their irritating faults. | 0.012 | 0.606 |
| SC04 | Most older adults are not different from anybody else: they are as easy to understand as young people | 0.041 | 0.613 |
| SC07 | Most older adults would prefer to quit work as soon as their children can support them | 0.041 | 0.621 |
| SC05 | Most older adults are set in their ways and unable to change | 0.048 | 0.625 |
| SC23 | There are a few exceptions, but most older adults are the same | 0.060 | 0.630 |
| SC31 | Most older adults make excessive demands for love and reassurance | 0.021 | 0.637 |
| SC17 | Most older adults spend much time prying into the affairs of others | 0.055 | 0.644 |
| SC03 | There is something different about most older people: it is hard to figure out what makes them different | 0.042 | 0.653 |

SC= Section C of questionnaire

**Table 5. Final items of KAOP**

|  | **Items** | **Corrected Item-Total Correlation** | **Cronbach's Alpha if Item Deleted** |
| --- | --- | --- | --- |
| SC02 | It would be better if most older adults lived in housing that also housed younge | 0.246 | 0.640 |
| SC06 | Most older adults are capable of new adjustment when the situation demands it | 0.285 | 0.636 |
| SC08 | Most older adults would prefer to continue working as long as they can, rather than be dependent on others | 0.251 | 0.640 |
| SC10 | Most older adults maintain a clean attractive home | 0.409 | 0.625 |
| SC12 | People grow wiser with age | 0.171 | 0.648 |
| SC14 | Most older adults are very easy to be with | 0.309 | 0.632 |
| SC16 | Most older adults past expereinces are interesting | 0.346 | 0.631 |
| SC18 | Most older adults respect others privacy and give advice only when asked | 0.289 | 0.635 |
| SC20 | Most older adults have same faults as any body else | 0.143 | 0.652 |
| SC22 | A nice neighbourhood is one that has a number of old people living in it | 0.223 | 0.642 |
| SC24 | Most older adults are very different from one another | 0.131 | 0.651 |
| SC26 | Most older adults are quiet clean in their personal appearances | 0.369 | 0.628 |
| SC28 | Most older adults are cheerful and good-humoured | 0.215 | 0.643 |
| SC30 | Most older adults seldom complain about the younger generation's behaviours | 0.153 | 0.651 |
| SC32 | Most older adults need the same amount of love and reassurance as anyone else | 0.234 | 0.641 |
| SC09 | Most older adults tend to let their homes become messy and unattractive | 0.225 | 0.642 |
| SC10 | It is foolish to claim that wisdom comes with age | 0.142 | 0.653 |
| SC13 | Most older adults make people uncomfortable | 0.180 | 0.647 |
| SC15 | Most older adults bore others by talking about the good old days | 0.132 | 0.653 |
| SC21 | In order to maintain a nice neighbourhood, it would be best if older adults did not live in it | 0.184 | 0.646 |
| SC25 | Most older adults are untidy in their personal appearances | 0.146 | 0.650 |
| SC27 | Most older adults are irritable and unpleasant | 0.294 | 0.634 |

SC= Section C of questionnaire
